# Emotional intelligence and academic performance in first and final year medical students: a cross-sectional study

**DOI:** 10.1186/1472-6920-13-44

**Published:** 2013-03-27

**Authors:** Boon How Chew, Azhar Md Zain, Faezah Hassan

**Affiliations:** 1 Department of Family Medicine, Faculty of Medicine & Health Sciences, Universiti Putra Malaysia, 43400 Serdang, Selangor, Malaysia; 2 Medical Education Unit, Faculty of Medicine and Health Sciences, Universiti Putra Malaysia, Selangor, Malaysia; 3 Department of Psychiatry, Universiti Putra Malaysia, Selangor, Malaysia

**Keywords:** Emotional intelligence, Educational assessments, Achievement, Medical students

## Abstract

**Background:**

Research on emotional intelligence (EI) suggests that it is associated with more pro-social behavior, better academic performance and improved empathy towards patients. In medical education and clinical practice, EI has been related to higher academic achievement and improved doctor-patient relationships. This study examined the effect of EI on academic performance in first- and final-year medical students in Malaysia.

**Methods:**

This was a cross-sectional study using an objectively-scored measure of EI, the Mayer-Salovey-Caruso Emotional Intelligence Test (MSCEIT). Academic performance of medical school students was measured using continuous assessment (CA) and final examination (FE) results. The first- and final-year students were invited to participate during their second semester. Students answered a paper-based demographic questionnaire and completed the online MSCEIT on their own. Relationships between the total MSCEIT score to academic performance were examined using multivariate analyses.

**Results:**

A total of 163 (84 year one and 79 year five) medical students participated (response rate of 66.0%). The gender and ethnic distribution were representative of the student population. The total EI score was a predictor of good overall CA (OR 1.01), a negative predictor of poor result in overall CA (OR 0.97), a predictor of the good overall FE result (OR 1.07) and was significantly related to the final-year FE marks (adjusted R^2^ = 0.43).

**Conclusions:**

Medical students who were more emotionally intelligent performed better in both the continuous assessments and the final professional examination. Therefore, it is possible that emotional skill development may enhance medical students’ academic performance.

## Background

Studies have found that emotional intelligence (EI) is related to academic and professional success and contributes to individual cognitive-based performance over and above the level attributable to general intelligence [1]. People and college students with higher emotional intelligence show more positive social functioning in interpersonal relationship and are regarded by peers as prosocial, less antagonistic and conflictual [2]. These improved social competence and quality relationships could facilitate cognitive and intellectual development leading to better academic performance [3-5]. In a more direct way, EI facilitates prioritizing of thoughts, behaviour regulation and appropriately adapted lifestyle choices which benefits academic performance [2]. Emotional intelligence, one of the psycho-affective domains, in medical education, has also been related to clinical performance and higher academic achievement [6]; and in clinical practice, has been related to improved empathy in medical consultation, doctor-patient relationships, clinical performance and patient satisfaction [7-10]. EI is defined as the ability to perceive emotions, to access and generate emotions so as to assist thought, to understand emotions and emotional meanings, and to reflectively regulate emotions so as to promote both better emotional and intellectual growth [11]. This ability model of EI posits four related skills: perceiving emotions, using emotions to facilitate thinking, understanding emotions, and managing emotions.

There is an urgent need today to inculcate the virtues of patient-care and self-care in healthcare professionals [12-14]. Patients need more than what a purely analytical doctor can offer. Recovery and therapeutic processes for patients could be more effective with a doctor who was communicating empathetically, ethically and competently [14,15]. These professional attributes are well enshrined in the Fundamentals of Professionalism- the physician charter by the ABIM Foundation, Foundation of Medical Ethics and Principles of Biomedical Ethics [16,17]. Demand for a physician who is genuinely interested in the health of patients, incorporating a patient’s personal values and engaging with them in health decisions is only increasing in an ageing society, and one with more educated patients and higher standards of healthcare [18]. With these changing external demands and internal quality requirements, it is not hard to comprehend that today’s physician has to be both smart and “good” [19]. The former generally refers to cognitive knowledge and technical skills, while the latter infers being virtuous and resilient, both mentally and emotionally. The latter quality has increasingly been recognized as equally, if not more important, and could well contribute to the former [20-22]. Thus, many medical school programs for prospective students often look into these personal qualities as part of their admissions process [23-25]. Henceforth, the medical education continuum from the medical schools to continuing professional/medical education is working hard to see that these other traits and skills are being assessed, maintained and developed [26-29].

However, there currently exist little data on the effect of EI on academic performance in medical education. This is partly due to the evolving of the concept in the last decades and the availability of various psychometric measures [30]. With different approaches to defining and measuring EI there were inconsistent findings regarding the relationship between EI and academic performance [1,7,31]. For example, many have used self-reports of constructs such as mood, optimism and motivation [32]. The Emotional Competence Inventory (ECI) and Bar-On Emotional Quotient Inventory (EQi) are among the popular self-report EI measures which were found lacking in discriminant validity in terms of potential overlap with personality traits, and most importantly, the lack of an ability or performance based component [1].

Accordingly, we used the ability-based instrument Mayer-Salovey-Caruso Emotional Intelligence Test (MSCEIT) to measure EI for predicting academic performance in medical education. For this purpose, we examined the effect of EI in first- and final-year undergraduate medical students attending a public medical school in Malaysia. First-year medical students were chosen because of their transition period from home to independent living in the college with a new learning environment. Final-year medical students are facing the impending professional examination with conferment of a doctor degree and internship in the same calendar year, and this period is considered another high-stress time for this group of students. Compared to other years’ medical students, the first- and final-year medical students are considered to be in more emotion-demanding academic years and thus the effect of EI on academic performance would be more apparent.

## Methods

This study was approved by the Universiti Putra Malaysia (UPM) Medical Research Ethics Committee. This was a cross-sectional study using the MSCEIT as the instrument to measure emotional intelligence.

### Participants

The participants were the first- and final-year medical students 18 years old or older pursuing an undergraduate degree. Students who refused to participate, who were ever diagnosed to have psychiatric disorders, or who did not understand English, were excluded from the study.

### Academic performance

Academic performance measures consisted of the total continuous assessment (CA) and the final examination (FE) marks, in averaged percentages with marks range from 0 to 100%. We used these as our primary study outcomes because they were important objective summative assessments. Scores on these tests decide whether the first-year students would proceed to the second year; and the final-year students to be conferred the medical degree. The first-year continuous assessment comprised the average percentage of the total of five end-of-package examinations on basic medical sciences. The final-year continuous assessment comprised the average percentage of the total six surgical and medical postings. These end-of-posting assessments comprise written assessment (multiple choice questions) and clinical examinations (long-case and/or short-case clinical examination). The final examination marks for first-year students was the end of the Package 6 examination. Final examination marks for final-year students were the aggregate Professional Examination III, which consists of multiple choice questions (MCQ), modified essay questions (MEQ), objective structured clinical examination (OSCE), long-case and short-case clinical examination. The first-year Package 1 to Package 6 examinations comprise written assessment (multiple choice and short answer questions) and objective structured practical examinations (OSPE). In the written exams, the skills required are mainly cognitive and affective. In the practical exams such as OSPE, OSCE, short-case and long-case clinical exams, the skills would involve more of psychomotor besides affective and cognitive domains. Performance was coded using the standard A to F grading system, with the following cut scores for each letter grade: A ≥ 75%, B + =70–74%, B 65–69%, B- 60–64%, C 50–59% and F < 50%.

### The emotional intelligence assessment

The MSCEIT is designed to measure the four branches of the EI model of Mayer and Salovey for adults aged 18 years and older [11,33]. MSCEIT was developed from an intelligence-testing tradition formed by the emerging scientific understanding of emotions and their function. Although self-report measures of EI are commonly used, self-assessments of EI most likely reflect perceptions of emotional abilities rather than measuring the abilities themselves. Furthermore, many self-report measures of EI overlap significantly with common personality measures, whereas the MSCEIT has demonstrated minimal overlap with personality [34,35]. Unlike traditional self-report tests, the MSCEIT asks participants to engage in the types of tasks that are thought to employ the four abilities of EI. For example, to assess the first branch, perceiving emotions, respondents are shown pictures of faces displaying varying degrees of emotion and are asked to rate the level of various emotions. MSCEIT consists of 141 items and takes 30–45 minutes to complete by self-administration. Two scoring keys were developed, one based on the general consensus of the normative sample of 5,000 people and the other on the consensus of a group of 21 international emotions experts. The expert key was employed in this study as this is more in line with an ability measure. Responses on the MSCEIT are scored with respect to their degree of correctness, as determined by their correspondence with the answers provided by the group of emotions experts. The overall reliability of the test is r = 0.91 for expert scoring. MSCEIT is both content and structurally valid, besides showing discriminate validity from measures of analytic intelligence and many personality constructs [11].

**Figure 1:**
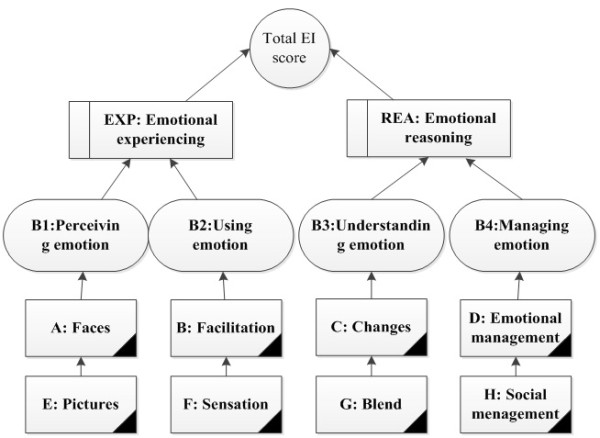
The MSCEIT scores.

MSCEIT provides 15 scores (Figure 1): Total EI score (TOT), two Area scores (EXP: emotional experiencing, REA: emotional reasoning), four Branch scores (B1: perceiving emotion, B2: using emotion, B3: understanding emotion, B4: managing emotion), and eight Task scores (A: faces, B: facilitation, C: changes, D: emotion management. E: pictures, F: sensation, G: blends, H: social management). Faces Task asks respondents to identify feelings based upon facial expressions. Facilitation Task measures respondents’ knowledge interaction of moods and thinking. Changes Task measures respondents’ knowledge of emotional “chains”, the possible emotions transition in given situations. Emotion Management Task measures the ability of incorporating one’s emotions into decision making. Pictures Task requires respondents to indicate various emotions that are being expressed in images and landscapes. Sensations Task requires the respondents to link emotions to different sensations such as light, colours and other sensory modalities. Blends Task measures respondents’ ability in analysing mixture of emotions and dissembling complex feelings into parts of simple emotions. Lastly, the Social Management Task measures the respondents’ ability to incorporate emotions into decision making that involves other people [33]. Further descriptions of the Branch and Area score have been published elsewhere [33]. We report on the results of the Total score only as some studies have questioned the four-factor structure of the MSCEIT [2,11,33,34].

The MSCEIT was administered in English, and given that not all students were native speakers, a glossary of some of the more difficult vocabulary words explaining them with simpler English words and/or synonyms was provided to study participants.

### Other study variables

The other study variables, designed to measure critical aspects of medical education important in Malaysian medical schools, were included in a one-page paper form. The items were: age, gender (male or female), ethnicity of student based on their paternal side (Malay, Chinese, Indian, Aborigine or other), total income of all family members of parents and siblings in Ringgit Malaysia (<2 000, 2 000–4 999, 5 000–9 999, 10 000–19 999 and >20 000), and having a medical doctor in the family (first and second degree relatives, from grandparents to children of the siblings). A series of questions examined self-perceived support from family (encouragement, financial support, etc.) in pursuing the study of medicine, self-perceived decision to study medicine based on family or personal choice, extent of social life while on campus (including hostels, hospitals etc.), enjoyment in studying medicine, anxiety (feelings of distress and overwhelmed) and religiosity (in adhering to one’s religion’s requirement and ways of life). These questions employed a five point Likert scale (very satisfied to very unsatisfied). The last two items were about perceptions of the teaching facility (availability and experiences in utilising teaching and learning facilities in the residing campus and/or hospital) and teacher quality (teacher’s ability to deliver course materials). These responses were recorded on a five-point Likert scale from very satisfied to very unsatisfied. These variables were chosen for their possible effect on academic performance in medical schools [36].

The questionnaires were pilot tested with 12, year four medical students for clarity and acceptability and minor modifications were made to increase clarity.

### The setting

The first- and final-year medical students were contacted in the second semester. The timing was such so that EI assessment was close to the final examination. A briefing on the study was held in their respective classes, and the information sheet and consent form were distributed. Those that agreed to participate by returning the signed consent form were scheduled in batches according to the number of available computers with internet access in the medical faculty’s computer laboratories. Students completed the MSCEIT online, followed by the demographic questionnaire. Each student was identified by their student number and they were allowed to receive their score results if they indicated so in the questionnaire. Examination results were obtained from the academic office.

### Statistical analyses

Data were analysed using Statistical Package for the Social Sciences (SPSS) version 19. Independent variables were the demographic parameters and the total MSCEIT scores while the dependent variables were the student’s assessment marks and grades. Some of the demographic variables were dichotomised and coded as a “1” as follows: female, high income (> RM 10 000), have a doctor in the family, good family support (very good and good ratings), self-intention to study medicine (disagree and very disagree ratings), socialize well in the campus (very agree and agree ratings), enjoy studying medicine (very agree and agree ratings), feeling anxious (disagree and very disagree ratings), religiosity (very agree and agree ratings), satisfaction with teaching facility and teacher quality, both were (very satisfied and satisfied ratings). Good and poor overall academic performances were defined as examination marks ≥ 70% and < 60%, respectively. For the final-year medical students, we used ≥ 65% as the cut-off for good academic performance. These cut-offs were used mainly to allow adequate sample sizes for analyses and yet remain meaningful in terms of their impact on promotion or granting of the degree.

The independence assumption and multicolinearity were checked and satisfied. Histogram and normal probability plots were used to check on the normality assumptions of the residual values.

The continuous variables were tested for statistical significance using the Student’s t-test or ANOVA (analysis of variance) while the Chi-square test was utilised for categorical variables. Tests of significance were two-tailed, and a *p* value of less than 0.05 was considered as statistically significant. Bivariate analyses were performed to identify the significant associated factors for multivariate analysis. Gender was entered in all the multivariate analyses because of its significant relationship with EI in many previous studies [7,37]. We reported the Nagelkerke R Square and the Adjusted R Square, from multiple logistic regression and multiple linear regression respectively, as the variance in marks or grades explained by each of the model. We checked the results with different groupings of the responses and found consistent results.

## Results

A total of 163 medical students participated in the study, a response rate of 66.0%. They were 84 (out of 122) medical students in the first-year and 79 (out of 125) in the final-year (Table 1). The gender and ethnic distribution were representative of the student population [38]. Final-year medical students had generally similar MSCEIT (TOT, Area, Branch and Task) scores compared to the first-year medical students except that students in the final-year scored better in the Understanding Branch (B3) (t-test: 4.44, p = 0.04) and a specific component of this branch score, Task C, the Changes task (t-test: 6.89, p = 0.01). Of all of the socio-demographic factors, ethnicity was the only one that had significant association with both the CA and FE. Medical students of Malay ethnicity were generally performing more poorly when compared to students of other ethnicities in CA (ANOVA: F_3,158_ = 31.21, P < 0.0001) and FE (ANOVA:F_3,158_ = 20.96, P < 0.0001).

**Table 1:** Bio-demography for the year 1 ancd year 5 medical students

	**n (%)**			**Mean (SD)**	
	**All**	**First year**	**Final year**	**CA**	**FE**
**Age**, mean (SD)	21.8 (1.98)	20.0 (0.50)^*^	23.8 (0.59)^*^	NA	NA
**Gender**					
Female	112 (68.7)	58 (69.0)	54 (68.4)	61.3 (6.72)	62.7 (6.83)
Male	51 (31.3)	26 (31.0)	25 (31.6)	60.4 (8.18)	61.7 (8.97)
Total	163 (100)	84 (100)	79 (100)	61.0 (7.19)	62.4 (7.54)
**Ethnicity**					
Malay	85 (52.1)	48 (57.1)	37 (46.8)	***57.0 (5.50)***^‡^	***58.7 (6.52)***^‡^
Chinese	63 (38.7)	29 (34.5)	34 (43.0)	***66.0 (6.08)***^‡^	***66.9 (6.32)***^‡^
Indian	11 (6.7)	5 (6.0)	6 (7.6)	***64.1 (4.47)***^‡^	***66.2 (5.92)***^‡^
Others	4 (2.5)	2 (2.4)	2 (2.5)	***58.7 (8.53)***^‡^	***60.3 (8.02)***^‡^
Total	163 (100)	84 (100)	79 (100)	61.0 (7.19)	62.4 (7.54)
**Gross Family Income**					
< 2,000	44 (28.0)	24 (29.3)	20 (26.7)	60.6 (8.31)	61.6 (8.69)
2,000–9,999	96 (61.1)	49 (59.8)	47 (62.7)	61.2 (7.06)	62.7 (7.24)
> 10,000–19,999	17 (10.8)	9 (10.9)	8 (10.7)	61.6 (5.60)	64.0 (6.59)
Total	157 (100)	82 (100)	75 (100)	61.1 (7.25)	62.5 (7.59)
**Have doctor/s in the family**					
Yes	40 (25.2)	21 (25.3)	19 (25.0)	***62.3 (6.62)***^‡^	63.7 (6.73)
No	114 (71.7)	59 (71.1)	55 (72.4)	***60.9 (7.30)***^‡^	62.2 (7.89)
Not Sure	5 (3.1)	3 (3.6)	2 (2.6)	***53.9 (7.08)***^‡^	58.2 (4.78)
Total	159 (100)	83 (100)	76 (100)	61.0 (7.23)	62.5 (7.57)
**Family Support**					
Very good/Good	147 (92.5)	73 (88.0)	74 (97.4)	61.3 (6.81)	62.6 (7.17)
Not sure	9 (5.7)	8 (9.6)	1 (1.3)	56.3 (12.58)	59.7 (13.24)
Very poor/Poor	3 (1.9)	2 (2.4)	1 (1.3)	60.0 (2.13)	65.8 (4.66)
Total	159 (100)	83 (100)	76 (100)	61.0 (7.23)	62.5 (7.57)
**Studying medicine is my family’s intention**					
Very agree/Agree	17 (10.7)	***11 (13.3)***^†^	***6 (7.9)***^†^	60.5 (6.51)	63.2 (5.96)
Not sure	20 (12.6)	***16 (19.3)***^†^	***4 (5.3)***^†^	58.7 (8.45)	61.7 (7.31)
Very disagree/Disagree	122 (76.7)	***56 (67.5)***^†^	***66 (86.8)***^†^	61.5 (7.09)	62.5 (7.85)
Total	159 (100)	83 (100)	76 (100)	61.0 (7.23)	62.5 (7.57)
**Socialize well in the campus**					
Very agree/Agree	120 (75.5)	57 (68.7)	63 (82.9)	61.0 (6.10)	62.3 (6.53)
Not sure	36 (22.6)	24 (28.9)	12 (15.8)	60.9 (10.39)	62.7 (10.59)
Very disagree/Disagree	3 (1.9)	2 (2.4)	1 (1.3)	62.2 (3.72)	64.6 (3.83)
Total	159 (100)	83 (100)	76 (100)	61.0 (7.23)	62.5 (7.57)
**Enjoying my study in medicine**					
Very agree/Agree	109 (68.6)	51 (61.4)	58 (76.3)	61.7 (6.76)	63.0 (7.34)
Not sure	37 (23.3)	24 (28.9)	13 (17.1)	59.0 (8.52)	60.8 (8.65)
Very disagree/Disagree	13 (8.2)	8 (9.6)	5 (6.6)	60.8 (6.41)	62.9 (5.77)
Total	159 (100)	83 (100)	76 (100)	61.0 (7.23)	62.5 (7.57)
**Feeling anxious most of the time**					
Very agree/Agree	70 (44.0)	36 (43.3)	34 (44.7)	***60.4 (6.70)***^‡^	62.1 (7.22)
Not sure	41 (25.8)	27 (32.5)	14 (18.4)	***59.2 (8.88)***^‡^	61.1 (9.08)
Very disagree/Disagree	48 (30.2)	20 (24.1)	28 (36.8)	***63.4 (5.83)***^‡^	64.1 (6.47)
Total	159 (100)	83 (100)	76 (100)	61.0 (7.23)	62.5 (7.57)
**I am a religious person**					
Very agree/Agree	117 (74.1)	66 (79.5)	51 (68.0)	60.7 (7.21)	62.5 (7.46)
Not sure	22 (13.9)	11 (13.3)	11 (14.7)	59.9 (7.65)	61.4 (6.70)
Very disagree/Disagree	19 (12.0)	6 (7.2)	13 (17.3)	63.8 (6.65)	63.1 (9.04)
Total	158 (100)	83 (100)	75 (100)	61.0 (7.25)	62.4 (7.57)
**Satisfaction on teaching facilities**					
Very satisfied/Satisfied	120 (75.5)	57 (68.7)	63 (82.9)	60.5 (7.31)	62.0 (7.90)
Not sure	13 (8.2)	8 (9.6)	5 (6.6)	62.4 (7.76)	63.5 (6.92)
Very unsatisfied/Unsatisfied	26 (16.4)	18 (21.7)	8 (10.5)	62.8 (6.43)	64.3 (6.10)
Total	159 (100)	83 (100)	76 (100)	61.0 (7.23)	62.5 (7.57)
**Satisfaction on teachers’ quality**					
Very satisfied/Satisfied	126 (79.2)	***58 (69.9)***^†^	***68 (89.5)***^†^	60.8 (6.90)	62.2 (7.16)
Not sure	15 (9.4)	***12 (14.5)***^†^	***3 (3.9)***^†^	59.1 (7.30)	60.7 (9.54)
Very unsatisfied/Unsatisfied	18 (11.3)	***13 (15.7)***^†^	***5 (6.6)***^†^	63.9 (8.90)	65.4 (8.23)
**Total**	159 (100)	83 (100)	76 (100)	61.0 (7.23)	62.5 (7.57)

CA = Continuous Assessment, FE = Final Examination, NA = Not Applicable.

* Student’s t-test with P value < 0.05.

^†^ Chi-square test with P value < 0.05.

^‡^ ANOVA with P value < 0.05.

A correlation analysis between all of the MSCEIT scores and the overall results in CA and FE is reported in Table 2. There were more significant correlations between MSCEIT (TOT, Area, Branch and Task) scores and the overall CA results than for the overall FE results; the TOT score was correlated with overall CA score (r = 0.24) and overall FE score (r = 0.21) (Table 2). When the association of TOT and academic performance was examined separately in each academic years, the significant correlation between TOT and CA was only observed in first-year medical students (r = 0.29, p = 0.01), and the significant correlation between TOT and FE was only observed in final-year medical students (r = 0.28, p = 0.02).

**Table 2:** Correlation between MSCEIT scores and the overall academic performance, n = 162

	**Continuous assessment**		**Final exam**	
	**r**	p*	**r**	p*
**Total EI score**	0.24	**0.003**	0.21	**0.01**
**Two Area scores**				
1. Experiential EI score	0.23	**0.003**	0.21	**0.01**
2. Strategic EI score	0.18	**0.02**	0.14	0.07
**Four Branch scores**				
1. Perceiving Emotions score	0.22	**0.01**	0.22	**0.01**
2. Facilitating Thinking score	0.17	**0.03**	0.11	0.18
3. Understanding Emotion score	0.24	**0.002**	0.18	**0.02**
4. Emotional Management score	0.02	0.81	0.04	0.61
**Eight Task scores**				
1. Face Task score	0.20	**0.01**	0.25	**0.002**
2. Pictures Task score	0.12	0.14	0.04	0.59
3. Sensations Task score	0.09	0.28	0.04	0.61
4. Facilitation Task score	0.25	**0.002**	0.17	**0.03**
5. Blends Task score	0.24	**0.002**	0.21	**0.01**
6. Changes Task score	0.18	**0.03**	0.10	0.23
7. Emotion Management Task score	0.07	0.40	0.09	0.27
8. Emotional Relations Task score	−0.02	0.82	−0.02	0.84

*Pearson’s correlation.

### EI and overall academic performance

Because gender and native language have an impact on the emotional intelligence scores, we conducted multiple regression analyses on CA and FE results. After adjusting for gender, ethnicity as well as socialization, the TOT score was still a predictor of a good result in the CA (Table 3). These predictors accounted for 32.3% of the variance of good CA result. After adjusting for gender, ethnicity and teacher quality, the TOT score was still a predictor of a good result in the FE (OR 1.07 95% CI 1.019 to 1.116). This model could explain 37.8% of the variation in achieving good FE results. After adjusting for gender, ethnicity, intention of study, self-perceived anxiety and religiosity, the TOT score was a negative predictor of achieving a poor result in CA (OR 0.97 95% CI 0.935 to 1.000). This model could explain 47.3% of the variation in achieving poor CA result.

**Table 3:** Predictors for good result in continuous assessment (marks ≥ 70%) in both the first year and final year medical students, n = 155

	OR*	**95% CI**	**p value**
**Total MSCEIT**	1.056	1.005, 1.110	**0.030**
**Gender**			
Male	1	-	-
Female	0.655	0.177, 2.418	0.525
**Ethnicity**			
Malay	1	-	-
Chinese	21.466	2.554, 180.406	**0.005**
Indian	10.035	0.533, 189.097	0.124
**Socialization**			
Socialize well & unsure	1	-	-
Not socialize well	2.093	0.711, 6.166	0.180

*Multivariate Logistic Regression using Enter Method.

### EI and final-year academic performance

Multiple linear regression shows that female, non-Malay student and the TOT score (B = 0.11 95% CI 0.027 to 0.190, p = 0.010) were significantly related to the final-year FE marks (adjusted R^2^ = 0.43, F_6,64_ = 9.76, P < 0.0001) (Table 4). After adjusting for gender and ethnicity, the TOT score remained a predictor of fairly good results (marks ≥ 65%) in CA (OR 1.06 95% CI 1.004 to 1.121). This model could explain 41.6% of the variation in achieving a good CA result. After adjusting for gender, ethnicity and socialization, the TOT score still remained a predictor of fairly good results (marks ≥ 65%) in the FE (OR 1.09 95% CI 1.019 to 1.155). This model could explain 55.1% of the variation in achieving a good FE result.

**Table 4:** Multiple regression model for final year FE with gender, ethnicity, having doctor in the family, socialization, self-reported anxiety and total MSCEIT score, n = 71

**Model**	**Unstandardized coefficients**		**Standardized coefficients**	**t**	**Sig.**	**95.0% Confidence interval for B**	
	B*	**Std. error**	**Beta**			**Lower bound**	**Upper bound**
(Constant)	47.331	3.435		13.778	.000	40.468	54.193
Female (1)	2.325	1.056	.207	2.201	**.031**	.215	4.436
Chinese and Indian (1)	6.245	1.022	.588	6.108	**.000**	4.202	8.287
Doctor in the family (1)	.909	1.208	.074	.753	.454	−1.504	3.323
Not socialize well (1)	1.985	4.356	.044	.456	.650	−6.716	10.687
Not anxious (1)	1.929	1.030	.172	1.872	.066	−.129	3.986
Total MSCEIT	.109	.041	.251	2.650	**.010**	.027	.190

*Multiple Linear Regression used Enter Method.

FE = Final Examination.

## Discussion

We found that EI was a significant predictor of academic performance in overall continuous assessments and final examination amongst first- and final-year medical students in a Malaysian university. This result could indicate the significant presence of a direct EI effect on academic performance in medical education. The EI influence on academic performance seemed mainly due to students’ ability to accurately perceive emotions and to their ability to understand emotional causes. This knowledge would enable the students with higher EI to have more adaptive life-styles, be more attuned to the signals of others and themselves, and better understand the causes of their and others’ emotions [11,39]. The lack of correlation between EI and the first-year FE could be due to the fact that FE is simply the end-of-Package 6 examination, compared to an accumulation of five examinations in the CA or the more extensive FE in the final year. It was also possible that the first-year medical students were experiencing less negative emotion or that the stress and strain of study were somehow buffered by the positive feeling coming from the fact of them being admitted into a medical school. In addition, higher EI would not have been as necessary in the first-year students who might be less concerned with study because studying medicine was not their own intention or who had been overly disappointed with teacher’s quality (see Table 1) [11]. In final-year medical students, the weak correlation of EI and CA was probably due to the students’ familiarity with the learning activity of postings rotation through the different surgical and medical specialties and thus less emotion-laden in facing the end-of-posting CA. In addition, the final-year medical students would have become more mature and experienced from their previous clinical years of facing small end-of-posting examinations. Therefore, the influence of EI in facing CA would be less apparent compared to when facing the FE which involves more tests and spans over several days [40].

The total EI score was related mainly to the final-year medical students’ academic performance but not to the performance of first-year students. This result might be due to the fact that this group was more heterogeneous (see Table 1) as compared to the final-year group. It is possible that the English proficiency might be poorer or more varied in the first-year students leading to inconsistent MSCEIT results. These different levels of English proficiency were observed from our experience in teaching these two groups of students. This study found that the first- and final-year students had generally similar MSCEIT scores except that final year students scored better in Branch 3, Understanding emotion, and more specifically, Task C (Changes). In another study in the United Kingdom that used MSCEIT, a similar observation was reported that older medical students scored significantly higher in the Understanding emotion branch [41]. The other possible explanation would be that it is more emotionally demanding in the final year of study which has its academic curriculum packed with many varied clinical postings and the students are constantly preparing for the final professional examination. In this high emotional period of study, EI could have its effect and impact on academic performance. This EI effect in emotion demanding situation was reported by Joseph who observed that EI positively predicts performance for high emotional labor jobs [40].

More generally, the small effect size of EI versus the large influence of demographic variables such as ethnicity is an important finding. Although the effect size is small, it was significant beyond gender and ethnicity. Ethnicity, in this study, can also serve as a proxy variable for English language fluency. That is, although the language of instruction in this Malaysian medical school is English, and all students take an English-language proficiency examination as part of the admissions process, students with different ethnic backgrounds likely have differing levels of English-language fluency. Given that the MSCEIT was given in English and uses emotion terms typically not covered in language exams, some of the effects for EI may be due to language rather than emotion ability.

Our findings were in line with the general expectation of a positive relationship between emotional intelligence and academic performance. Austin et al. reported a significant correlation between EI and students’ performance in problem-based learning sessions but no correlation was found between EI and end-of-year examination marks [31]. However, that study employed a self-report EI scale in a sample of 100 end-of-year examination marks from medical students in year 1, 2 and 5, so it is difficult to make meaningful comparisons with our present study. People with higher EI are expected to understand, regulate and manage emotions better both in themselves and others. This would allow people with higher EI to experience more stable and positive emotions. Medical students with positive attitude and emotion towards their course were related to academic achievement [42]. In this study, second year medical students in the US who self-reported to have higher course-related enjoyment had higher scores on The National Board of Medical Examiners (NBME) shelf examination (β = 0.31) in contrast to their classmates who were reported to have course-related anxiety and boredom (β = − 0.36 and - 0.27, respectively) [42]. Furguson et al. reported that medical students in Nottingham who scored high on emotional stability (assessed by the big five personality domains) performed better on three out of eight pre-clinical assessments under their study [43]. Jaeger et al. reported that for students in a general management graduate-level course, greater levels of emotional intelligence (using a self-report measure) correlated with academic performance (final project grade comprised a paper and oral presentation) even when controlling for traditional markers of intelligence [44]. This study had also provided evidence that EI training incorporated within the curriculum could improve the students’ EI to the extent of having a positive effect on the academic performance.

The present study reported a positive impact of emotional intelligence on academic performance among Asian medical students with a multicultural background. With the report of high levels of stress and anxiety among the medical students and the positive effect of EI on academic performance in this and other studies [41], future prospective and intervention studies with emotional training programs are needed to confirm these findings [45,46]. More work is also necessary to find factors underlying academic performance which have the potential to be trained. The effect of such training might not only improve the students’ academic performance, in terms of examination marks, but also their experience of the teaching and learning activities [47]. Doctors with more developed emotional intelligence skills could also provide more quality patient-centred care [48], cope better with their highly demanding professional career and perhaps lead a more fulfilled and happier life as an individual [7,8,49].

### Limitations

This study was one of the few that employed an ability-based measure of emotional intelligence instead of self-report surveys. Our results add to growing research showing influence of EI, in ability-type EI measures, on academic performance in an undergraduate medical program. However, there were several limitations of this study. The first limitation is the sample size within each academic year. Second, selection bias could play a role, as in any voluntary study; those non-participating students could be less motivated or discouraged with their already poor academic achievement. Third, the EI measure was administered in English and not all subjects were native English speakers. The effect of English proficiency on the MSCEIT and academic performance in this study cannot be ascertained. As this confounding effect of English proficiency is more likely in the first-year medical students, future work could include the students’ Malaysian University English Test (MUET) results that they are required to sit for in the first semester in the first year. Some of the items in the MSCEIT use scenarios surrounding family or personal issues which could be unfamiliar to the students. Since the objective of this study was to examine the effect of EI in undergraduate medical students on academic performance, we did not report the contribution of general intelligence towards the examination results and the relationship of general intelligence and EI. Admission into medical schools is competitive in Malaysia as well as elsewhere; where only the high achievers from the pre-university or matriculation programs are admitted. Thus, it was believed that there would be a limited range of general intelligence among the medical students at this university. Because of the more demanding medical degree program with reports of increasing prevalence of emotional disorders among medical students [38,50-54], the results reported here are hoped to draw attention to the effect of EI on academic performance. Future work could examine the relationship of general intelligence and EI, and their combined effect on the academic performance in medical education.

## Conclusions

Medical students who were more emotionally intelligent performed better in both the continuous assessments and the final professional examination. The independent effect of EI on academic performance was more prevalent in final-year final examination marks. EI predicts only a small amount of variance, but it does so beyond gender and ethnicity. Medical schools may want to examine the emotional intelligence of its students, and possibly, to provide greater emotional skill development to medical students to enhance learning and academic performance.

## Competing interest

The authors declare that they have no competing interests.

## Authors’ contributions

CBH, FH and AMZ contributed to the conception and design of the study. CBH and FH contributed to the acquisition, analysis and interpretation of data. CBH contributed to statistical analysis and drafted the manuscript. All authors contributed to the critical revision of the manuscript. All authors read and approved the final manuscript.

## Pre-publication history

The pre-publication history for this paper can be accessed here:

http://www.biomedcentral.com/1472-6920/13/44/prepub

## Previous presentations

Oral presentation in the 19th Wonca Asia Pacific Regional Conference in Jeju, Korea from March 24th to 27th, 2012.
